# Evaluation of herpesvirus members on hospital admission in patients with systemic lupus erythematous shows higher frequency of Epstein-Barr virus and its associated renal dysfunction

**DOI:** 10.1590/2175-8239-JBN-2021-0184

**Published:** 2022-04-04

**Authors:** Katia Lino, Lilian Santos Alves, Natalia Trizzotti, Jessica Vasques Raposo, Cintia Fernandes Souza, Andrea Alice da Silva, Vanessa Salete de Paula, Jorge Reis Almeida

**Affiliations:** 1 Universidade Federal Fluminense, Hospital Universitário Antônio Pedro, Niterói, RJ, Brasil.; 2 Universidade Federal Fluminense, Laboratório Multiusuário de Apoio à Pesquisa em Nefrologia e Ciências Médicas, Niterói, RJ, Brasil.; 3 Universidade Federal Fluminense, Departamento de Medicina Clínica, Niterói, RJ, Brasil.; 4 Universidade Federal Fluminense, Departamento de Patologia, Niterói, RJ, Brasil.; 5 Universidade Federal Fluminense, Programa de Pós-graduação em Ciências Médicas, Niterói, RJ, Brasil.; 6 Universidade Federal Fluminense, Faculdade de Medicina, Programa de Pós-Graduação em Patologia, Niterói, RJ, Brasil; 7 Fundação Oswaldo Cruz, Instituto Oswaldo Cruz, Laboratório de Virologia Molecular, RJ, Brasil.

**Keywords:** Herpesviridae, Lupus Erythematosus, Systemic, Herpesvirus 4, Human, Creatinine, Lupus Nephritis, HSV-1, CMV, Kidney, Herpesviridae, Lúpus Eritematoso Sistêmico, Herpesvirus Humano 4, Creatinina, Nefrite Lúpica, VHS-1, CMV, Rim

## Abstract

**Introduction:**

Members of the Herpesviridae family have been described in patients with systemic lupus erythematous (SLE), but the clinical impact on renal function is not well known.

**Methods:**

HSV1, HSV2, VZV, EBV, CMV, HHV-6, HHV-7, and HHV-8 were evaluated by molecular biology on admission in blood samples from 40 consecutive SLE patients hospitalized for lupus activity.

**Results:**

Patients were 90.0% female, 77.5% non-white, with average age of 32.7 ± 13.6 years. We found positivity for EBV (65.0%), CMV (30.0%), HSV-1 (30.0%), HHV-6 (12.5%), and HHV-7 (7.5%). For all viruses, age, SLEDAI, hematological tests, ferritin, LDH, C-reactive protein, and erythrocyte sedimentation rate (ESR) were not significant. However, EBV positivity was a significant factor for higher serum creatinine (3.0 ± 2.8 vs. 0.9 ± 0.8; P = 0.001) and urea (86 ± 51 vs. 50 ± 46; P = 0.03). Moreover, positive cases for EBV only or with combined co-infections (66.7%-CMV; 58.3%-HSV-1) or negative for EBV only were evaluated by Kruskal-Wallis test again showed statistical significance for serum creatinine and urea (both P ≤ 0.01), with posttest also showing statistical differences for renal dysfunction and EBV presence (alone or in combined co-infections). The presence of EBV viral load was also significant for nephrotic-range proteinuria, renal flare, and the need for hemodialysis.

**Conclusion:**

Members of the Herpeviridae family (mainly EBV, HSV-1 and CMV) are common on hospital admission of SLE patients, reaching 65% for EBV, which seems to be associated with renal dysfunction and could reflect a previous association or overlapping disease, which is not well understood.

## Resumo

**Introdução:**

Membros da família Herpesviridae tem sido descritos em pacientes com lúpus eritematoso sistêmico (LES), mas o impacto clínico na função renal não é bem conhecido.

**Métodos:**

Avaliou-se HSV1, HSV2, VZV, EBV, CMV, HHV-6, HHV-7, HHV-8 por biologia molecular na admissão em amostras sanguíneas de 40 pacientes com LES consecutivos hospitalizados por atividade lúpica.

**Resultados:**

Pacientes 90,0% mulheres, 77,5% não brancos, idade média 32,7 ± 13,6 anos. Encontramos positividade para EBV (65,0%), CMV (30,0%), HSV-1 (30,0%), HHV-6 (12,5%), HHV-7 (7,5%). Para todos os vírus, idade, SLEDAI, exames hematológicos, ferritina, LDH, proteína C reativa, velocidade de hemossedimentação não foram significativos. Entretanto, positividade para EBV foi estatisticamente significativo para creatinina (3,0 ± 2,8 vs. 0,9 ± 0,8; P = 0,001) e ureia (86 ± 51 vs. 50 ± 46; P = 0,03) séricas mais elevadas. Ademais, casos positivos para EBV isolado ou com coinfecções combinadas (66,7%-CMV; 58,3%-HSV-1) ou negativos apenas para EBV foram avaliados pelo teste Kruskal-Wallis e novamente mostraram significância estatística para creatinina e ureia séricas (ambas P ≤ 0,01), com pós-teste mostrando também diferenças estatísticas para disfunção renal e presença de EBV (sozinho ou em coinfecções combinadas). A presença de carga viral do EBV também foi significativa para proteinúria de faixa nefrótica, inflamação aguda, necessidade de hemodiálise.

**Conclusão:**

Membros da família Herpeviridae (principalmente EBV, HSV-1, CMV) são comuns na admissão de pacientes com LES, chegando a 65% para EBV, que parece associar-se à disfunção renal podendo refletir associação prévia ou doença sobreposta, o que não é bem compreendido.

## Introduction

The human herpesvirus family may cause severe diseases in immunocompromised individuals, such as those with autoimmune diseases, cancer and transplant patients^1,2^. This family includes the following viruses: herpes simplex virus 1 and 2 (HSV-1 and HSV-2), varicella-zoster virus (VZV), Epstein-Barr virus (EBV), cytomegalovirus (CMV), human herpes virus 6 and 7 (HHV-6 and HHV-7), and the Kaposi’s sarcoma-associated herpesvirus 8 (HHV-8)^2^. Herpesviruses have been reported in case series of SLE patients as an emergent issue, including triggering of SLE itself and its activity, through viral reactivation mechanisms and immune-inflammatory disturbance^3^. Recently, a relationship between SLE, chronic kidney disease, and EBV load has been suggested^4^. Thus, the aim of this cross-sectional pilot study was to assess the frequency of infection with each human herpesvirus and the possible clinical association with kidney disease on hospital admission of systemic lupus erythematous (SLE) patients.

## Methods

This was a retrospective laboratory-based study. The presence of all members of the human herpesvirus family was assessed in the same blood sample from each consecutive adult SLE patient on hospital admission, regardless of gender, who was hospitalized for investigating febrile status associated with SLE activity. The study was performed at the Antonio Pedro University Hospital in Niteroi, Brazil, from June, 2019 to January, 2020. The blood samples were collected in the first 24 hours of admission. Samples were immediately processed to obtain serum for biochemical analysis. Samples for hematological testing and total blood aliquot were collected in anticoagulant EDTA tubes, immediately stored at-80º Celsius, and used for DNA extraction (QIAamp DNA Minikit, Qiagen, Germany). We excluded patients diagnosed with cancer, HIV, syphilis, viral hepatitis, pregnancy, and SLE transplanted patients. The study was approved by the Ethics Committee (CAAE: 12125219.8.0000.5243).

The herpesvirus family members were studied using in-house methods in the Laboratory of Molecular Virology (Oswaldo Cruz Foundation, Rio de Janeiro), in which the viral load was evaluated by PCR amplification performed in a 7500 system (Applied BiosystemsTM, Foster City, CA, USA) using specific primers and probes. The detection limits of qPCR were 10 copies/mL according to previous methodology^5-7^. Biochemical and hematological analysis were performed at a local clinical pathology unit. SLE was classified by an experienced assistant rheumatologist (K.L.) in the first 3 days after hospital admission, according to the Systemic Lupus International Collaborating Clinics (SLICC)^8^, and SLE activity was estimated using the SLEDAI 2K scores^9^.

Serum creatinine was assessed on admission and plotted against the presence of herpesvirus. From outpatient medical records, we checked serum creatinine and urea up to 3 months before the day of admission. This allowed us to classify the cases as recent renal dysfunction or not and verify if creatinine values were stably high up to 3 months before the day of admission, in which this case the patient was considered to have chronic kidney disease. Proteinuria was assessed on the day of admission and up to 48 hours after using urine collected for 24 hours or by urinary protein/creatinine ratio in a morning sample, with cases being arbitrarily divided into three classes: less than 1g/day, more than 1g/day, and nephrotic-range proteinuria (more than 3.5g/day). For practical considerations of a real-life study, the term renal flare (lupus nephritis activity) was used in this paper to refer to both newly diagnosed lupus or lupus occurring during the course of disease characterized by a significant increase in serum creatinine or the appearance/worsening of proteinuria with fever, increased serum inflammatory markers, active urinary sediment (if present), and complement uptake. On the other hand, we also considered recent and unexplained increase in serum creatinine of at least 0.5 mg/dL or more than 30% of the basal level when associated with dehydration, gastrointestinal loses, other hypovolemic status, heart failure, direct drug toxicity, tubulointerstitial nephritis and sepsis (if not ruled out by the lupus itself), even when kidney biopsies were not available. In the context of lupus, a composite including these situations was used as a categorical variable to evaluate renal outcomes in these cases. Data were expressed as mean and standard deviation. Differences between groups were assessed using Mann-Whitney and Kruskal-Wallis tests. Analyses were performed with SPSS and Prism Graphpad statistical packages. We considered a P value < 0.05 as significant.

## Results

The presence of human herpesvirus 1 to 8 family members was evaluated by molecular biology in blood samples from the first day of hospitalization in 40 consecutive patients with SLE hospitalized due a clinical picture of lupus activity. No positivity for HSV-2, VZV and HHV-8 was observed in the blood samples, but we found 26 samples positive for EBV (65.0%), 12 for CMV (30.0%), 12 for HSV-1 (30.0%), 5 for HHV-6 (12.5%), and finally 3 for HHV-7 (7.5%). The majority of patients (90.0%) were female with average age of 32.7 ± 13.6 years, predominantly non-white (77.5%). The median of SLE duration was 6.0 years. Most of the patients (60.0%) presented SLEDAI 2K scores ≥ 4. Table 1 summarizes the general characteristics of the patients.

**Table 1:** General characteristics of hospitalized SLE patients (n = 40) and the presence of herpesviridae family members

Age; years, mean ± SD	32.7 ± 13.6
Skin Color; non-White, n (%)	31 (77.5 %)
Gender; % Female, n (%)	36 (90.0 %)
SLE time; years, median (95% CI)	5.5 (4.6 - 6.9)
SLEDAI 2K; scores, mean ± SD	5.0 ± 4.2
SLEDAI 2K ≥ 4; n (%)	24 (60.0 %)
Lymphopenia (<1000 cells/mm3); %	52.9 %
Erythrocyte sedimentation rate; mm/h, mean ± SD	79.7 ± 46.2
C Reactive protein; mg/dL, mean ± SD	5.9 ± 9.9
HSV-1; n (%)	12 (30.0 %)
EBV; n (%)	26 (65.0 %)
CMV; n (%)	12 (30.0 %)
HHV-6; n (%)	5 (12.5 %)
HHV-7; n (%)	3 (7.5 %)

Abbreviations: HVS-1: herpes simplex virus 1; HSV-2: herpes simplex virus 2, VZV: varicella-zoster virus, EBV: Epstein-Barr virus, CMV: cytomegalovirus, HHV-6: human herpes virus 6, HHV-7: human herpes virus 7, HHV-8: Kaposi’s sarcoma-associated herpesvirus 8.

SLE: systemic lupus erythematous. HSV-2, VZV and HHV-8 were not detected.

Age, SLE duration, SLEDAI, leukocytes, hemoglobin, platelets, lymphocytes LDH, ferritin, ESR, and CRP were all not significant for any herpes positivity. However, SLE patients with EBV positivity were significantly associated with higher levels of serum creatinine (3.0 ± 2.8 vs. 0.9 ± 0.8; P = 0.001) and urea (86 ± 51 vs. 50 ± 46; P = 0.03). Table 2 summarizes these findings for EBV, CMV, and HSV-1. In addition to the lower levels of glomerular filtration rate, EBV-positive patients presented a tendency to be older and have longer SLE duration, associated with tendency of higher levels of ESR, CRP, and LDH, but not significantly.

**Table 2:** Clinical and laboratory correlations on hospital admission of SLE patients (total = 40) according to blood presence of herpes virus HSV-1, CMV, and EBV

	HSV-1 (n = 12)			CMV (n = 12)			EBV (n = 26)		
Parameters	NEG	POS	P	NEG	POS	P	NEG	POS	P
Age, years	33.2 ± 14.2	31.6 ± 12.8	0.73	31.7 ± 14.5	34.9 ± 11.6	0.47	29.1 ± 13.7	34.6 ± 13.5	0.23
Time of SLE, months	78 ± 92	97 ± 48	0.45	88 ± 86	70 ± 51	0.50	62 ± 54	100 ± 92	0.14
SLEDAI 2K, scores	5.6 ± 4.7	5.7 ± 2.8	0.96	6.0 ± 4.3	4.7 ± 4.0	0.35	6.0 ± 4.3	5.4 ± 4.3	0.69
Leukocytes, cells/mm^3^	6,779 ± 6,132	7,214 ± 2,,439	0.75	6,685 ± 5,294	7,483 ± 5,229	0.68	6,785 ± 3,319	6,984 ± 6,037	0.90
Hemoglobin, g/dL	8.4 ± 1.5	7.7 ± 2.3	0.37	8.3 ± 1.9	7.8 ± 1.5	0.45	8.5 ± 1.0	8.0 ± 1.7	0.46
Platelets, 10^3^/mm^3^	190 ± 118	204 ± 116	0.74	215 ± 125	147 ± 77	0.05	208 ± 150	188 ± 96	0.67
Lymphocytes, cells/mm^3^	1,171 ± 892	994 ± 1,122	0.64	994 ± 811	1,419 ± 1,275	0.35	1,194 ± 1,056	1,060 ± 919	0.70
LDH, U/L	423 ± 266	336 ± 158	0.32	370 ± 213	456 ± 296	0.50	325 ± 119	436 ± 281	0.18
Ferritin, ng/mL	1012 ± 716	652 ± 715	0.27	866 ± 754	945 ± 682	0.82	858 ± 699	909 ± 765	0.87
ESR; mm/h	68 ± 50	96 ± 38	0.14	83 ± 48	72 ± 44	0.59	66 ± 37	91 ± 51	0.16
CRP, mg/dL	7.1 ± 11.4	3.3 ± 5.0	0.35	5.8 ± 11.2	6.7 ± 5.0	0.82	2.9 ± 4.0	7.6 ± 12.0	0.25
Serum urea, mg/dL	77 ± 55	64 ± 41	0.42	72 ± 55	76 ± 46	0.77	50 ± 46	86 ± 51	0.03
Serum creatinine, mg/dL	2.2 ± 2.5	2.5 ± 2.5	0.70	2.3 ± 2.7	2.3 ± 1.8	1.00	0.9 ± 0.8	3.0 ± 2.8	0.00
Proteinuria ≥ 1g/day, yes/no (% of yes)	13/15 (46.4%)	6/6 (50.0%)	1.00	12/16 (42.9%)	7/5 (58.3%)	0.49	3/11 (21.4%)	16/26 (61.5%)	0.02
Nephrotic proteinuria, yes/no (% of yes)	10/18 (35.7%)	4/8 (33.3%)	1.00	9/19 (32.1%)	5/7 (41.7%)	0.72	0/14 (0.0%)	14/12 (53.8%)	0.00
Renal flare, yes/no (% of yes)	12/16 (42.9%)	6/6 (50.0%)	0.74	11/17 (39.3%)	7/5 (58.3%)	0.31	1/13 (7.1%)	17/9 (65.4%)	0.00
Hemodialysis, yes/no (% of yes)	9/19 (32.1%)	6/6 (50.0%)	0.59	9/19 (32.1%)	6/6 (50.0%)	0.31	2/12 (14.3%)	13/13 (50.0%)	0.04

Data are expressed by mean ± SD or %; (n) = number; P = Mann-Whitney test for continuous variable and chi-square test for categorical one; LDH = lactate dehydrogenase; CRP = C-reactive protein; ESR = erythrocyte sedimentation rate; HSV-1 = herpes simplex virus 1; EBV = Epstein-Barr virus; CMV = cytomegalovirus, SLE = systemic lupus erythematous.

No patient presented more than 3 herpesvirus types. Almost half of the patients (17/40, 42.5%) were under immunosuppressive therapy on hospital admission using prednisone at the usual maintenance doses associated with another immunosuppressant (AZA, MMF and MTX) and had more than 1 herpesvirus (7/17; 41.2%) with a significant frequency of EBV viral load (12/17; 70.5%). There were also patients using only prednisone (7/40; 17.5%), of which 3/7 (42.8%) had positive viral load for more than one herpesvirus, but with a lower incidence of EBV viral load (2/7; 28.5%). A group with no immunosuppressants (recent SLE diagnosis, non-adherence to treatment, or with no prescription) (11/40; 27.5%) had 6/10 (60%) positive cases for more than 1 herpesvirus, and 9/11 (82.0%) were EBV-positive. A small number of patients (using only MMF/AZA (2/40)) or prednisone and cyclophosphamide (2/40)) did not contribute to this analysis (AZA/MMF = 1 EBV each). We provide this information in more detailed in Table S1.

Table S1 also provides detailed clinical description of the patients on admission, as well as some clinical outcomes. Table 2 shows significant relationships between EBV with proteinuria, renal flare, and hemodialysis. We found no significant relationship with mechanical ventilation and death. In Table S1 we provide also serum urea and creatinine values up to 3 months before admission, and that shows that the majority of cases are in fact acute renal dysfunction, regardless of the age or gender.

**Figure 1:**
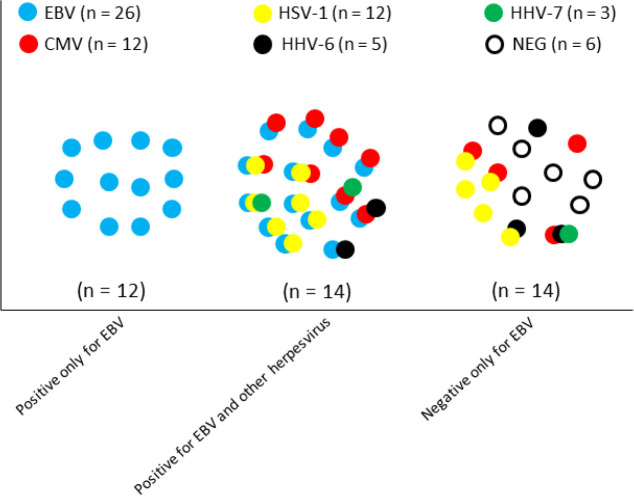
Schematic diagram of hospital admission of SLE patients (n = 40) and distribution of herpesvirus types and detectable viral load (n = 58). The colors and overlapped circles represent different viruses and patients, respectively. This grouping helps to identify patient clusters positive only for EBV (blue circles), positive for EBV and other herpesviruses (multicolored circles), and negative only for EBV (without blue circles). HSV-1 = herpes simplex virus 1, EBV = Epstein-Barr virus, CMV = cytomegalovirus, HHV-6 = human herpes virus 6, and HHV-7 = human herpes virus 7. This diagram helps with the analysis of Figure 2.

**Figure 2:**
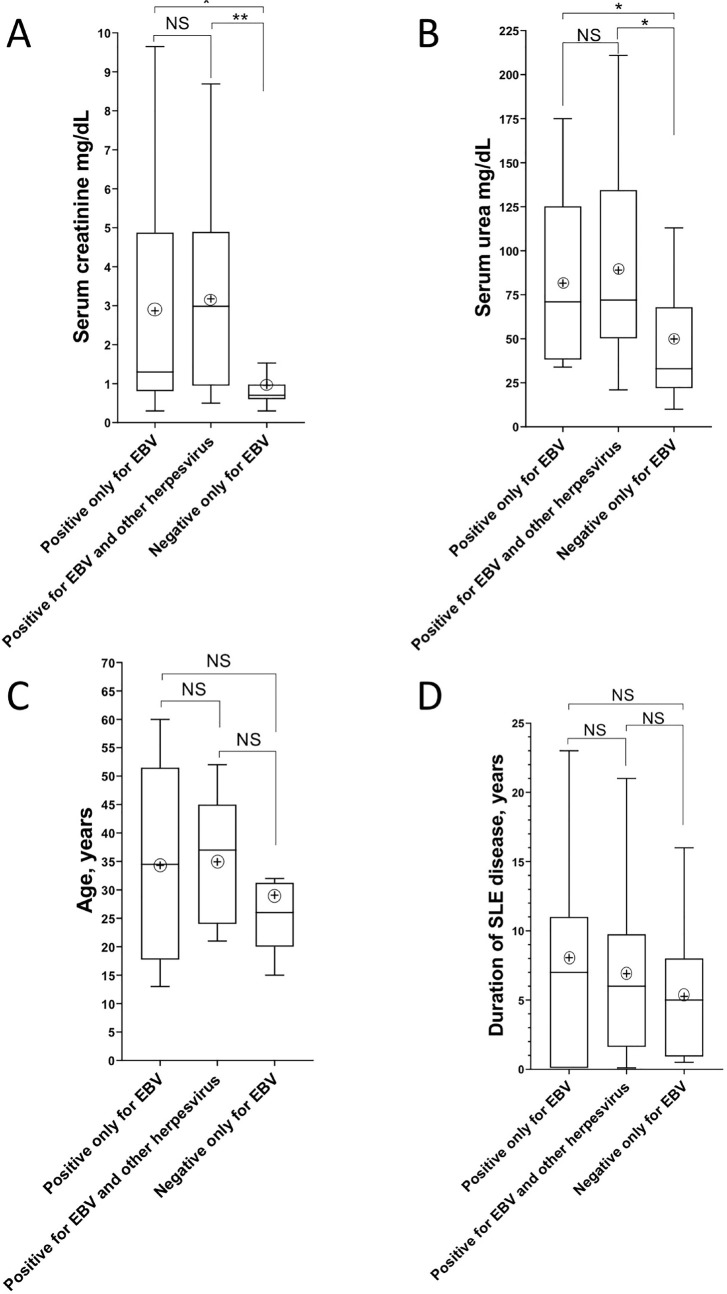
Representative box plot of Kruskal-Wallis test for serum creatinine and urea, according to EBV clusters seen in Figure 1. For serum creatinine (A) and urea (B), P ≤ 0.01 and 0.03 were found, respectively. Differences between pairs were evaluated with post-tests and represented by stars and bars, *≤ 0.05, and **≤ 0.01. NS: not significant. The box plots represent medians, minimum, and maximum values. Circled crosses represent the mean in each box. Correspondent age (C) and duration of SLE disease (E) are represented at the bottom and were non-significant.

In total, we found 58 infections. Patients with at least one infection were 85%, while patients with any combination of co-infections were 45%. Besides, EBV had the highest frequency of cases; EBV alone represented almost half of the cases (46%) and when associated to other virus, it accounted for 54%. It is also noteworthy that the majority of CMV and HSV-1 cases had combined co-infection with EBV. Figure 1 helps to understand the distribution of the cases and it reveals three clusters of further interest: positive only for EBV (blue circles), positive for EBV and other herpesvirus in combined co-infection (multicolor circles), and cases negative only for EBV (without blue circles). In this way, we tested according with this clustering design for the same clinical and laboratorial variables. According to the Kruskal-Wallis test, the only significant factors was serum creatinine and urea (P=0.01). The post-tests showed statistical differences for EBV alone and in combined co-infection groups for serum creatinine and serum urea. Figure 2 shows more detailed results.

Of the total, 18/40 (45%) presented with renal flare, of which 16/18 (88.9%) were positive for EBV. Patients diagnosed with chronic kidney disease were 6/40 (15%), of which 5/6 (83.3%) were also positive for EBV. Of note, 13/40 (32.5%) presented nephrotic-range proteinuria and all of them (13/13; 100%) were positive for EBV. Among patients with proteinuria ≤ 1g/day (21/40; 52.5%), only 10/21 (47.6%) were positive for EBV. Patients with no renal dysfunction were 16/40 (40%), of which only 5/16 (31.2%) were positive for EBV. Positive cases for EBV had significantly more indication for hemodialysis. Renal flare and proteinuria were not significant in patients with and without HVS-1 and CMV viral load. Table 2 summarizes these findings.

Of note, on admission, 10/40 (25%) of the patients had blood culture positive mostly for Staphylococcus species, *E. coli*, and Candida species, with no significant correlation with EBV frequency or renal flare (Table S1).

## Discussion

We found positivity for HSV-1 and CMV and an impressive frequency of 65.0% for EBV in hospitalized SLE patients. Infections are common in patients with SLE, resulting partly from immunosuppressive treatment and immune system disruption associated with SLE and are associated with high morbidity and mortality^10^. The identification and correlation of active human herpesvirus with clinical complications in SLE patients with high SLEDAI scores is very important, as is the early search for members of the herpes family such as the CMV in the first days of hospitalization of patients with autoimmune diseases^3,11^. For example, subjects with SLE might exhibit increased susceptibility for EBV and/or CMV and HHV-6 reactivation with or without implications in disease exacerbation^12^. Among the herpesvirus family, EBV has been often implicated in SLE pathogenesis and considered a potential trigger for SLE flares, with patients showing evidence of increased EBV reactivation^13^. EBV-specific CD8+ T cell response are functionally impaired in SLE patients, but EBV reactivation appears to be an aggravating consequence, which could contribute to the perpetuation of immune activation^14,15^.

The most important point of our work emerges when we associate positivity for the three most frequent viruses found (EBV, HSV-1, and CMV) with several clinical variables including age, SLEDAI scores, biochemical, and hematological tests (**Table 2**). We did not find any evidence of clinical impact, except for statistical significance between serum creatinine and urea values and positivity for EBV and also for proteinuria, renal flare and the start of hemodialysis. We studied SLE patients who had relatively uniform clinical activity (SLEDAI 2K ≥ 4 of 60%, lymphopenia, and high ESR levels) (Table 1), and despite lacking a control group, our series could be of interest due to the relative homogeneity in a real-life study. To better certify and understand the relationships of EBV viral load and renal dysfunction, we studied the distribution of the virus in patients to identify subgroups according to the presence or absence of EBV, including EBV alone and combined co-infections (Figure 1). Thus, we were able to separately assess the impact of EBV and found a strong body of mathematical evidence that associated EBV infection with renal dysfunction, whether for EBV alone or in combined co-infections (Figure 2).

We found a strong association between EBV presence and renal outcomes in SLE hospitalized patients, including general renal dysfunction but especially the degree of proteinuria, renal flare, and need for hemodialysis. These findings are in agreement with some studies addressing the presence of EBV in renal tissue from patients with lupus nephritis^16^. EBV antigens are able to generate pathogenic antinuclear antibodies that cross-react with double-stranded DNA, causing glomerular immune deposition, proteinuria, and histopathological lesions of glomerulonephritis in experimental lupus nephritis^17^, as well as in small kidney biopsy using PCR for EBV, and in immunohistochemistry study for the severity of nephropathy^16,18^. However, despite no significant association with serum EBV antibodies, 100% positivity for anti-VCA-IgG-EBV was seen in SLE patients with renal manifestations^19^. Recently, a study compared SLE outpatients with healthy controls and found a relationship between high levels of viral load for EBV with chronic kidney disease and DNA fragmentation in the SLE group^4^. In this way, we state that without neglecting other immunological or infectious considerations, the presence of EBV could have a consistent impact on kidney damage and progression of kidney disease in SLE patients. However, the discussion about these points in terms of pathogenesis, including for example the over production of anti-Sm, molecular mimicry, or if EBV reactivation could occur even before the onset of lupus nephritis, or if there would be a proposal for how to approach and treat these cases, are beyond the scope of this brief communication. Nevertheless, these points deserve to be urgently studied.

Herpesvirus infection should always be considered in SLE patients with a wide range of clinical presentations, such as severe encephalitis, lymphoid activation, or pulmonary and gastrointestinal lesions, but this was beyond the scope of this study. A concomitant chronic state of viral subclinical replication and how to treat these cases should also be investigated.

In this study, the frequency of total detectable herpesviruses, especially EBV, in patients newly diagnosed for lupus or with flare due to non-adherence to treatment or even with no immunosuppressant prescription was similar to the group using prednisone plus AZA/MMF/MTX. That is in accordance with other studies pointing out that disease activity per se is associated with herpesvirus viral load, especially EBV^14^, in an independent manner, and that deserves more studies. On the other hand, we observed also a similar number of total cases of herpesvirus in patients using only prednisone, but it was associated with lower number of detectable EBV. These findings are not conclusive due to a wide range of prednisone doses used by our patients and the small number of cases.

Our work presents some relevant and original points. We studied all herpesvirus members at admission, more specifically EBV (alone or in co-infections), highlighting the pre-admission renal dysfunction and an important association with EBV. As this is a laboratory-based descriptive retrospective series, this study has some weaknesses such as the lack of an age- or sex-matched control group. However, in our opinion, the most important point is the fact that it suggests an association between renal dysfunction and the EBV viral load. It is also important to emphasize the need for well-designed future clinical studies, approaching SLE patients longitudinally by monitoring the viral load over time, controlling for activity index and evaluating early occurrence of lupus activity. We believe we should be very attentive and careful, but there are no large systematic studies evaluating these findings, which point to the need for larger prospective studies.

In conclusion, despite of the high frequency of herpesvirus infections, particularly EBV, HSV-1, and CMV in SLE hospitalized patients, including co-infection in half of the cases, it seems that EBV has a special role in kidney disease reactivation and is associated with pre-admission kidney dysfunction. EBV infection seems to be associated with renal flare, proteinuria, and general kidney dysfunction.

## Supplementary Material

The following online material is available for this article:

Table S1- Descriptive data of SLE patients according to analysis of serum creatinine and presence of detectable herpesviruses (particularly EBV) on the day of admission and some clinical outcomes.
