# Supplementary material for: Evaluation of herpesvirus members on hospital admission in patients with systemic lupus erythematous shows higher frequency of Epstein-Barr virus and its associated renal dysfunction
Source: J Bras Nefrol. 2022 Apr 4;44(4):490–7. doi: 10.1590/2175-8239-JBN-2021-0184 (PMC9838661; doi:10.1590/2175-8239-JBN-2021-0184)
Supplement: Supplementary file 1 [file 2175-8239-jbn-2021-0184-suppl1.pdf]

Braz. J. Nephrol. (J. Bras. Nefrol.) 2022.

DOI: https://doi.org/10.1590/2175-8239-JBN-2021-0184

Supplementary material to: "Evaluation of herpesvirus members on hospital admission in patients with systemic lupus erythematous patients shows higher frequency of Epstein-Barr virus and its associated renal dysfunction"

Table S1. Descriptive data of SLE patients according to analysis of serum creatinine and presence of detectable herpesviruses (particularly EBV) on the day of admission and some clinical outcomes.

Patient	Age, years	SLE duration, years	Gender	Main clinical manifestation on admission	Immunosuppression therapy before admission	Serum creatinine 3 months before admission (mg/dL)	Serum creatinine on admission (mg/dL)	Serum urea 3 months before admission (mg/dL)	Serum urea on admission mg/dL)	Proteinuria on admission (≥ 1g/day) *nephrotic range	Renal flare/ AKI/Active lupus nephritis	HSV-1 Viral load	EBV Viral load	CMV Viral load	HHV-6 Viral load	HHV-7 Viral load	Kidney Biopsy	RRT	Main immunosuppression therapy during admission	Blood Culture	Mechanical ventilation	Death
01	32.0	5.0	F	Bullous skin lesions, arthritis, sicca syndrome and central nervous system manifestations	Oral prednisone, HCQ	0.60	0.30	18.0	31.0	NO	NO	3.24E+04	NEG	8.75E+02	NEG	NEG	NO	NO	MP pulse 1g 3 days, MMF.	Mucoid Burkholderia cepacia complex	NO	NO
02	28.0	0.5	M	Pleural effusion, endocarditis, heart failure	Oral dexamethasone HCQ	0.80	0.70	53.0	28.0	NO	NO	NEG	NEG	NEG	NEG	NEG	NO	NO	IVIg, antibiotics	Coagulase-Negative Staphylococci	YES	NO
03	15.0	0.5	F	Hemolytic anemia, convulsion, pleural and pericardial effusion, kidney failure	Newly diagnosed SLE	NA	5.50	NA	175.0	YES*	YES	NEG	1.53E+04	NEG	NEG	NEG	NO	YES	MP pulse 1g 3 days, IVIg, CP	NEG	YES	YES
04	18.0	5.0	F	CNS manifestations, vasculitis, antiphospholipid syndrome (APS), macrophage activation syndrome (MAS)	Oral prednisone; Azathioprine HCQ Acetylsalicylic acid	0.55	0.60	25.00	19.00	YES	NO	NEG	NEG	NEG	NEG	NEG	Class IV LN	NO	MP pulse 1g 3 days, IVIg, CP	NEG	YES	YES
05	52.0	0.5	F	SLE diagnosis 6 months before admission, hospitalized due acute respiratory distress syndrome	Oral prednisone, MTX, HCQ	0.60	1.30	28.0	36.0	NO	YES	NEG	2.93E+05	9.08E+06	NEG	NEG	NO	NO	MP pulse 1g 3 days, empiric liposomal amphotericin B, vancomycin, trimetoprim-sulfametoxazol.	NEG	YES	NO

Patient	Age, years	SLE duration, years	Gender	Main clinical manifestation on admission	Immunosuppression therapy before admission	Serum creatinine 3 months before admission (mg/dL)	Serum creatinine on admission (mg/dL)	Serum urea 3 months before admission (mg/dL)	Serum urea on admission mg/dL)	Proteinuria on admission (≥ 1g/day) *nephrotic range	Renal flare/ AKI/Active lupus nephritis	HSV-1 Viral load	EBV Viral load	CMV Viral load	HHV-6 Viral load	HHV-7 Viral load	Kidney Biopsy	RRT	Main immunosuppression therapy during admission	Blood Culture	Mechanical ventilation	Death
06	30.0	7.0	F	Rectal and perianal ulcers, suspected Crohn's disease overlapped with SLE	Oral prednisone, MMF, HCQ	1.06	0.89	38.0	10.0	NO	NO	5.06E+04	NEG	NEG	NEG	NEG	Class IV LN	NO	MP pulse 1g 3 days, MMF	NEG	NO	NO
07	36.0	8.0	F	Seizures, psychosis, class III LN and lupus renal vasculopathy 3 years before admission	Oral prednisone, MMF, HCQ	2.27	9.65	83.00	108.00	YES*	YES	NEG	2.93E+04	NEG	NEG	NEG	NO	YES	MP pulse 1g 3 days, plasmapheresis, CP, chronic hemodialysis	Contaminated samples	NO	NO
08	54.0	3.0	F	Skin manifestations, leukopenia and low platelets, class IV LN 4 months before admission, pyelonephritis, (E. coli)	Oral prednisone, CP, HCQ	5.80	0.90	133.0	98.0	NO	NO	NEG	NEG	NEG	NEG	NEG	NO	NO	Oral prednisone tapering, CP dose adjustment	NEG	NO	NO
09	63.0	16.0	F	Pericarditis, sacral ulcer and fistula, previous lupus nephritis, hemolytic anemia, chronic kidney disease	Oral prednisone, HCQ CP	1.50	1.53	77.0	58.0	YES	NO	3.24E+04	NEG	NEG	NEG	NEG	NO	YES	MP pulse 1g 3 days, empiric antibiotics	NEG	YES	YES
10	30.0	8.0	F	Pleural effusion, temporal arteritis, cephalalgia	Oral prednisone, MMF, HCQ, CP	0.54	0.64	16.0	17.0	NO	NO	NEG	NEG	NEG	NEG	NEG	NO	NO	MP pulse 1g 3 days, cyclosporine	NEG	NO	NO
11	20.0	0.5	F	Skin manifestation with suspected cutaneous lymphoma, hepatosplenomegaly, disseminated lymphadenopathy, bilateral pulmonary infiltrate, MAS	Newly diagnosed SLE	NA	0.75	NA	42.0	NO	NO	NEG	2.31E+04	NEG	NEG	NEG	NO	NO	MP pulse 1g 3 days, IVIg, plasmapheresis, CP	NEG	YES	NO
12	20.0	0.5	F	MAS, Class III lupus nephritis, pleural and pericardial effusion, rectocolitis, Anti-CMV IgM positive	Oral prednisone, HCQ	0.88	1.22	27.0	35.00	YES	YES	NEG	NEG	2.95E+04	NEG	NEG	NO	NO	MP pulse 1g 3 days, IVIg, CP, gancyclovir, echinocandin, metronidazole	Candida species	NO	NO
13	37.0	14.0	F	Past treatment for class IV lupus nephritis, diarrhea, low platelets, acute myocarditis	Oral prednisone AZA HCQ	1.60	4.59	38.0	75.0	YES*	YES	1.62E+04	1.96E+04	NEG	NEG	NEG	NO	YES	MP pulse 1g/day 3x; IVIg; CP	NEG	NO	NO
14	24.0	9.0	F	Juvenile lupus, ten years of disease, severe lymphopenia (CD4 very low), pulmonary tuberculosis, skin sporotrichosis	Non-adherence to therapeutic regimens	1.20	1.10	50.00	55.00	NO	NO	4.04E+04	1.48E+04	NEG	NEG	NEG	NO	NO	IVIg, itraconazole, tuberculosis treatment, oral prednisone low doses, HCQ	NEG	NO	NO
15	33.0	0.5	F	Anemia, disseminated strongyloidiasis, high dose of oral corticosteroids, edema, intestinal losses, perianal fistula	High oral dose prednisone	NA	2.1	NA	77.0	YES	YES	NEG	3.03E+05	NEG	NEG	NEG	NO	NA	NA	NA	NO	NO
16	38.0	8.0	F	Arthralgia, dry eyes, lymphadenopathy, hemolytic anemia, leucopenia, migraine	Oral prednisone AZA HCQ	1.10	1.20	38	37	NO	NO	NEG	9.62E+04	NEG	NEG	NEG	NO	NO	Dose adjustments (oral	NEG	NO	NO

Patient	Age, years	SLE duration, years	Gender	Main clinical manifestation on admission	Immunosuppression therapy before admission	Serum creatinine 3 months before admission (mg/dL)	Serum creatinine on admission (mg/dL)	Serum urea 3 months before admission (mg/dL)	Serum urea on admission mg/dL)	Proteinuria on admission (≥ 1g/day) *nephrotic range	Renal flare/ AKI/Active lupus nephritis	HSV-1 Viral load	EBV Viral load	CMV Viral load	HHV-6 Viral load	HHV-7 Viral load	Kidney Biopsy	RRT	Main immunosuppression therapy during admission	Blood Culture	Mechanical ventilation	Death
																			prednisone, AZA)			
17	25.0	9.0	M	Juvenile lupus, uveitis, APS, chronic lower limbs ulcers, anemia and lymphopenia, lupus nephritis	Oral prednisone MMF HCQ	2.40	0.80	124.00	69.00	NO	NO	NEG	1.33E+04	5.92E+04	NEG	NEG	NO	NO	Anticoagulants; MP pulse 500mg 3 days, (Cryoglobulins NEG)	NEG	NO	NO
18	17.0	8.0	F	Frequently reactivations, levels of C4 complement persistently low, toxic epidermal necrolysis-like lupus erythematosus, use of nimesulide and vancomycin	Oral prednisone, MMF, HCQ	0.54	0.92	17.0	34.0	NO	YES	NEG	3.55E+04	NEG	NEG	NEG	Class III LN	NO	Support therapy, adjustment of MMF doses	NEG	NO	NO
19	21.0	10.0	F	Juvenile lupus, Lymphopenia, Subacute meningitis	Oral prednisone HCQ	0.60	0.70	36.0	25.0	NO	NO	2.51E+04	NEG	NEG	1.50E+04	NEG	NO	NO	MP pulse 1g 3days	NEG	NO	NO
20	20.0	8.0	F	Diffuse Alveolar hemorrhage, pleural effusion, sinusitis, mastoiditis, acute kidney injury	Oral prednisone, MMF, HCQ	1.30	3.43	80.00	113,00	NO	YES	4.89E+04	NEG	5.92E+04	NEG	NEG	NO	YES	MP pulse 1g 3 days, IVIg, plasmapheresis, CP, antibiotics	NEG	YES	YES
21	53.0	12.0	F	Fever of unknown origin, lymphopenia, severe anemia	Oral prednisone AZA HCQ	0.90	0.83	37.0	45.0	NO	NO	NEG	4.15E+04	NEG	NEG	NEG	NO	NO	MP pulse 1g 3 days, empiric antibiotics	NEG	NO	NO
22	24.0	0.5	F	Acute and severe skin manifestation (Stevens-Johnson syndrome/toxic epidermal necrolysis-like lupus erythematosus)	Oral prednisone	0.75	0.66	18.0	23.0	NO	NO	NEG	NEG	NEG	5.40E+03	NEG	NO	NO	MP pulse 1g 3 days, IVIg, rituximab	Methicillin-sensitive S. aureus	NO	NO
23	15.0	3.0	F	Juvenile Lupus, recurrent pleural and pericardial effusion, investigation for myocarditis suspected on magnetic resonance, Hospitalized for endomyocardial biopsy	Oral prednisone, AZA, HCQ	1.10	0.74	21.00	37.00	NO	NO	NEG	NEG	NEG	NEG	NEG	NO	NO	IV MP 2mg/Kg/day, MP pulse !g 3 days, CP	NEG	NO	NO
24	25.0	6.0	F	Refractory cutaneous-articular activity, pancreatitis, edema of the lower extremities	Oral prednisone, AZA, MTX/belimumab	0.83	0.80	55.0	76.0	YES*	YES	NEG	5.47E+04	NEG	NEG	NEG	Class IV LN	YES	MP 50 mg/day IV, HCQ, MMF	NEG	NO	NO
25	39.0	6.0	F	Lymphopenia, chronic recurrent diarrhea	Oral prednisone, AZA	0.70	1.00	28.00	34.00	NO	NO	4.31E+04	5.65E+07	NEG	NEG	NEG	NO	NO	MP 50 mg/day, AZA, HCQ, antibiotics, anti-parasitic drugs	NA	NO	NO

Patient	Age, years	SLE duration, years	Gender	Main clinical manifestation on admission	Immunosuppression therapy before admission	Serum creatinine 3 months before admission (mg/dL)	Serum creatinine on admission (mg/dL)	Serum urea 3 months before admission (mg/dL)	Serum urea on admission mg/dL	Proteinuria on admission (≥ 1g/day) *nephrotic range	Renal flare/ AKI/Active lupus nephritis	HSV-1 Viral load	EBV Viral load	CMV Viral load	HHV-6 Viral load	HHV-7 Viral load	Kidney Biopsy	RRT	Main immunosuppression therapy during admission	Blood Culture	Mechanical ventilation	Death
26	55.0	23.0	F	Prolonged fever of unknown origin, class IV LN, frequent renal flares, Hemolytic anemia, lymphopenia	Oral prednisone AZA	4.09	8.00	104.00	131.00	YES*	YES	NEG	1.10E+05	NEG	NEG	NEG	NO	YES	MP pulse 1g 3 days, CP, antibiotics	Escherichia coli	NO	NO
27	28.0	6.0	F	Chronic kidney disease, anemia, leukopenia, visual symptoms	Non-adherence	5.80	6.40	99.00	211.00	NO	NO	NEG	3.60E+05	NEG	9.98E+03	NEG	NO	YES	MP 2m/Kg/day	Staphylococcus epidermidis	YES	YES
28	13.0	0.5	F	Nephrotic syndrome, neurolupus, hemolytic anemia, pancytopenia, MAS	Newly diagnosed SLE	0.20	0.30	25.0	35.0	YES*	YES	NEG	2.72E+04	NEG	NEG	NEG	Class II LN	NO	Class II LN, MP 2mg/kg, MP pulse 1g 3 days, IVIg, plasmapheresis	NEG	NO	NO
29	21.0	6.0	F	Lupus in remission, fever, skin rash and cervical lymphadenopathy	HCQ	0.60	0.50	33.00	36.00	NO	NO	NEG	NEG	NEG	NEG	NEG	NO	NO	Empiric antibiotic, oral prednisone, good response	NEG	NO	NO
30	47.0	0.5	F	Recent SLE diagnosis, with renal failure, on hemodialysis, daily fever, cervical lymphadenopathy, leukocytosis	Newly diagnosed SLE	1.8	3.0	99.0	153.0	YES*	YES	NEG	1.03E+05	NEG	NEG	NEG	Class III LN	YES	MP pulse 500mg 3 days, CP, kidney biopsy high scores of chronicity, vascular chronic lesions (atherosclerosis), partial recover of function	NEG	NO	NO
31	60.0	31.0	F	APL, pancytopenia, complement consumption, poor response to immunosuppressants	Oral prednisone	1.0	1.4	29.0	66.0	NO	NO	NEG	1.91E+05	NEG	NEG	NEG	NO	NO	Anticoagulation, MMF	NEG	NO	NO
32	21.0	6.0	F	Active lupus nephritis	Oral prednisone MMF, HCQ	1.50	8.69	64.00	127.00	YES*	YES	4.05E+05	1.63E+05	NEG	NEG	NEG	Class IV LN	YES	MP pulse 1g 3 days, CP, Class IV LN	NEG	NO	NO
33	23.0	0.5	F	Butterfly rash, arthralgia, pleural effusion, severe renal dysfunction	Newly diagnosed SLE	NA	3.33	NA	132.00	YES*	YES	4.48E+04	1.40E+06	1.16E+05	NEG	NEG	Class IV NL	YES	MP pulse 1g/day 3x; Plasmapheresis; CP, Class IV LN, high chronicity scores, chronic hemodialysis	Gram positive species	YES	NO
34	45.0	6.0	F	Poor adherence, choroidopathy, convulsive crisis, fever of unknown origin, diarrhea, previously class IV LN, unsatisfactory therapeutic response	Oral prednisone, HCQ	1.10	4.43	56.00	86.00	YES*	YES	5.43E+04	3.80E+05	1.81E+04	NEG	NEG	Class IV LN	YES	MP pulse1g 3 days, APL; hemodialysis, vancomycin and meropenem	Staphylococcus hemolyticus	NO	NO

Patient	Age, years	SLE duration, years	Gender	Main clinical manifestation on admission	Immunosuppression therapy before admission	Serum creatinine 3 months before admission (mg/dL)	Serum creatinine on admission (mg/dL)	Serum urea 3 months before admission (mg/dL)	Serum urea on admission mg/dL)	Proteinuria on admission (≥ 1g/day) *nephrotic range	Renal flare/ AKI/Active lupus nephritis	HSV-1 Viral load	EBV Viral load	CMV Viral load	HHV-6 Viral load	HHV-7 Viral load	Kidney Biopsy	RRT	Main immunosuppression therapy during admission	Blood Culture	Mechanical ventilation	Death
35	51.0	21.0	M	Pulmonary sepsis, critically ill patient	Poor Adherence	6.2	5.8	136.0	142.0	YES	NO	NEG	1.80E+06	1.74E+04	NEG	NEG	NO	YES	MP pulse 1g 3 days	NA	YES	YES
36	37.0	5.0	F	Hospitalized due gastrointestinal manifestations and hemodynamic status, fever of unknown origin, MAS	Oral prednisone, MMF, HCQ	0.6	1.5	25.0	57.0	YES*	YES	NEG	3.24E+04	5.26E+04	NEG	7.19E+03	NO	NO	MP pulse 1g 3 days, IVIg	NEG	NO	NO
37	38.0	12.0	F	Lupus reactivation, under treating of pulmonary tuberculosis, pleural effusion., lymphopenia, fever without an origin, sepsis, chronic kidney disease	AZA, Rifampicin, isoniazid	4.30	3.00	104.00	58.00	YES*	YES	NEG	7.64E+04	7.90E+04	7.25E+04	NEG	Class IV LN	YES	Disseminated candidiasis; Amphotericin B, maintenance chronic hemodialysis, MMF	Candida species	NO	NO
38	31.0	1.0	F	Sjögren + lupus (persistent activity), hemoptysis, negative screening for tuberculosis	MMF	0.80	0.60	32.0	16.00	NO	NO	NEG	NEG	3.29E+04	4.57E+04	5.41E+04	NO	NO	Oral prednisone, MMF	NEG	NO	NO
39	45.0	0.5	F	Previously healthy, recent lupus diagnosis, with severe lymphopenia, renal failure and MAS	Newly SLE diagnosed	NA	2.97	NA	143.0	YES*	YES	NEG	2.57E+05	1.91E+06	NEG	NEG	NO	YES	MP pulse 1g x 3 days, IVIg, CP; hemodialysis	Escherichia coli	YES	YES
40	24.0	2.0	F	Poor adherence, edema, ascites, pleural effusion	Irregular low dose prednisone	NA	0.55	NA	21.0	YES*	YES	4.37E+03	7.21E+04	NEG	NEG	1.63E+04	NO	NO	MP 2mg/kg; MMF; HCQ	NEG	NO	NO

Shortened forms of words (alphabetical order): antiphospholipid syndrome (APS), azathioprine (AZA), cyclophosphamide (CP), Gancyclovir (GAN), hydroxychloroquine (HCQ), high-dose intravenous immunoglobulins (IVIg), mycophenolate mofetil (MMF), methotrexate (MTX), not available (NA) data, methylprednisolone (MP), Renal replacement therapy (RRT), Lupus Nephritis (LN), Macrophage activation syndrome (MAS), Systemic Lupus Erythematous (SLE). The herpesvirus: herpes simplex virus 1 (HSV-1), Epstein-Barr virus (EBV), cytomegalovirus (CMV), human herpes virus 6 (HHV-6) and human herpes virus 7 (HHV-7).
